# In Vivo Confocal Microscopy of the Cornea in Diagnosing Small Fibre Neuropathy: A Cross-Sectional Observational Study

**DOI:** 10.3390/diagnostics15172207

**Published:** 2025-08-30

**Authors:** David Petrovič, Ajla Mujnović, Adela Hammami, Tjaša Krašovec, Mojca Kirbiš, Spela Stunf Pukl

**Affiliations:** 1 Faculty of Medicine, University of Ljubljana, 1000 Ljubljana, Slovenia; 2 Eye Hospital, University Medical Centre Ljubljana, 1000 Ljubljana, Slovenia; 3 Institute of Neurophysiology, University Medical Centre Ljubljana, 1000 Ljubljana, Slovenia

**Keywords:** small fibre neuropathy, in vivo confocal microscopy, skin biopsy, idiopathic small fibre neuropathy, secondary small fibre neuropathy, corneal nerve fibre density, corneal nerve branch density

## Abstract

**Objectives:** The aim of this study was to assess the accuracy of corneal in vivo confocal microscopy (IVCM) in the diagnostic process of small fibre neuropathy (SFN) compared to skin biopsy. **Methods:** This cross-sectional observational study was performed at the Eye Hospital and Institute of Neurophysiology, University Medical Centre Ljubljana, and included 35 patients with a clinical picture of SFN. All patients underwent a neurological exam that included an SFN questionnaire, standard skin biopsy, and ophthalmological assessment, including corneal IVCM. **Results:** Skin biopsy confirmed SFN in 14/35 patients (40%). These patients had a significantly shorter corneal nerve fibre length (CNFL) compared to those with negative biopsy (13.67 ± 2.99 mm/mm^2^ vs. 16.27 ± 3.54 mm/mm^2^, *p* = 0.030), as well as reduced corneal nerve branch density (CNBD) (36.68 ± 14.68 branches/mm^2^ vs. 48.81 ± 17.83 branches/mm^2^, *p* = 0.042). CNFL reduction below the 5th percentile was proven in 13/35 patients, yielding 64.3% sensitivity (95% CI: 35.1–87.2%) and 80.9% specificity (95% CI: 58.1–94.6%) compared to skin biopsy. In idiopathic SFN, negative IVCM results aligned with negative biopsies in 90% (95% CI: 55.5–99.8%) of cases. Meanwhile, in secondary SFN, positive IVCM results detected evidence of peripheral neurodegeneration in an additional 27.3% (95% CI: 6–61%) with negative skin biopsy. **Conclusion:** CNFL reduction in corneal IVCM demonstrated significant diagnostic value for SFN. Since skin biopsy findings do not always correspond with IVCM findings, corneal IVCM could be applied as a complementary tool to standard skin biopsy rather than as a replacement. It might be additionally useful for detecting patchy pattern presentations of SFN, excluding neuropathy in idiopathic SFN, and detecting neuropathy in biopsy-negative secondary SFN. In patients with positive IVCM, it could also be used as a primary tool for follow-up monitoring.

## 1. Introduction

The role of corneal in vivo confocal microscopy (IVCM) in the diagnostic process of small fibre neuropathy (SFN) is based on the possibility of non-invasive immediate insight into the morphology of small fibre corneal nerves through analysis and quantification. Despite several reports in the literature, IVCM is still not part of the routine diagnostic palette; therefore, its definitive diagnostic value is not generally accepted.

Small fibre neuropathy (SFN) is a syndromic disorder and encompasses a diverse group of neurological disorders characterized by neuropathic pain symptoms and autonomic complaints resulting from the selective involvement of thinly myelinated Aδ-fibres and unmyelinated C-fibres [1]. SFN can develop due to autoimmune, inflammatory, metabolic, toxic, and hereditary causes, while approximately 40% of cases are idiopathic [2,3]. SFN presents with a diverse range of clinical symptoms, positive or negative sensory phenomena, and autonomic dysfunction. An epidemiological study in the Netherlands showed an incidence of 12 cases per 100,000 inhabitants per year and a prevalence of 53 cases per 100,000 inhabitants [4].

Establishing the diagnosis is often challenging due to diverse clinical presentations and accompanying subjective pain reports with possible psychological or psychiatric issues. SFN cannot be confirmed by conventional nerve conduction studies, which can detect the dysfunction of large, unmyelinated fibres. Recent consensus on SFN diagnostic criteria confirms SFN when at least one SFN symptom and at least one clinical sign are present, corroborated by one of the confirmatory tests: skin biopsy for quantification of intraepidermal nerve fibre density (IENFD) or quantitative sensory testing (QST) [5,6,7].

Evaluation of intraepidermal small fibre density (IENFD) with skin biopsy is currently the most reliable diagnostic tool for SFN, with high accuracy, sensitivity, and specificity [8], but it has several drawbacks due to its invasiveness and time-consuming nature [9]. Moreover, the results do not consistently correlate with the severity of symptoms [10,11], and there are possible misdiagnoses due to fixative or histological stain problems. And last but not least, the so-called “patchy manifestation pattern” may result in missing small fibre changes in skin biopsy, as small fibre alterations can occur at various time points in different organs [12].

In vivo confocal microscopy (IVCM), on the other hand, allows for fast, non-invasive, in vivo imaging of corneal nerves with high resolution and possible repetition [13]. The cornea contains Aδ and unmyelinated C-class nerve fibres, which are characteristically affected in SFN [1,14]. Repeated analysis of the same part of the cornea enables longitudinal assessment of disease progression or evaluation of the effectiveness of therapy. It can also detect the underlying autoimmune and inflammatory process by visualizing dendritic cell morphology and density [15,16]. For the diagnosis of diabetic neuropathy, IVCM, compared to a skin biopsy, has a slightly higher sensitivity (0.77 vs. 0.61) and comparable specificity (0.79 vs. 0.80) [16]. Therefore, IVCM may potentially supplement skin biopsies and quantitative sensory testing (QST) as a standard diagnostic test in select patients.

Despite encouraging findings in the published literature, the precise diagnostic value of corneal IVCM in SFN patients is still unknown. The current diagnostic algorithm for SFN does not incorporate corneal IVCM. Trying to fill in this knowledge gap, we evaluated the relationship between skin biopsy results, corneal IVCM parameters, and different clinical symptoms and signs of neuropathy in a cross-sectional study of patients with a clinical picture of SFN. The purpose of the study was to assess the diagnostic value of corneal IVCM as compared to the standardized skin biopsy and to investigate its possible applications in SFN evaluation.

## 2. Materials and Methods

This was a cross-sectional observational study performed from September 2024 to March 2025 at the tertiary Eye Hospital University Medical Centre Ljubljana, Slovenia, and Institute of Neurophysiology, University Medical Centre Ljubljana, Slovenia.

### 2.1. Subjects

The patients were first examined by a neurologist, who clinically suspected a diagnosis of small fibre neuropathy (SFN). They were invited to participate in the study. Inclusion criteria included a clinical picture of SFN: at least 1 characteristic symptom (such as burning pain, prickling, sensation of cold or stabbing pain, autonomic dysfunction) and at least 1 positive clinical sign (such as pinprick and thermal hypoesthesia, autonomic skin changes), absence of electrophysiological dysfunction of efferent and large afferent fibres, absence of central nervous system disorders, 18 years or older, performed skin biopsy, and willingness to participate in the study and to sign the informed consent. Exclusion criteria included history of ocular trauma, history of corneal surgery or laser corneal prescription correction, history of intraocular surgery, any known ocular disease that affects corneal innervation (such as neurotrophic keratitis), and contact lens wear [17,18,19,20,21].

The sample size reflected all consecutive eligible patients within the study window who agreed to additional ophthalmological exam. To reduce selection bias, all patients were consecutively recruited from the neurology clinic based on predefined criteria. We acknowledge the referral bias, since patients were recruited from a tertiary centre, which may not represent the general SFN population. Both skin biopsy and corneal nerve analysis were interpreted against normative values. The invasive nature of the skin biopsy justifies the decision not to perform this in healthy candidates. However, we compared the symptomatic participants who had negative (normal) skin biopsy results to those with positive (reduced IENFD as compared to the normative database) skin biopsy results.

All participants who agreed to participate in the study signed the informed consent form.

### 2.2. Ethical Statement

This study was approved by the National Medical Ethics Committee of Slovenia (number 0120-477/2024-2711-3) and was performed following the Declaration of Helsinki.

### 2.3. Questionnaires

The participants included in the study were asked to fill out two questionnaires to assess the SFN symptoms: (i) the Neuropathic Pain Scale (NPS) questionnaire (scale X to Y, with higher numbers indicating more severe pain), and (ii) the Orthostatic Hypotension Questionnaire (OHQ) [22,23].

Supplementary Materials: English versions of NPS and OHQ.

### 2.4. Skin Biopsy

Standardized skin biopsy was performed to evaluate the intraepidermal nerve fibre density (IENFD) as part of the diagnostic process in all participants following the European Federation of Neurological Societies and the Peripheral Nerve Society guidelines [24].

IENFD was quantified from a 3 mm punch biopsy obtained 10 cm above the lateral malleolus, with local anaesthesia, using a 3 mm disposable punch with sterile technique. The samples were immunoassayed with PGP 9.5 antibody to improve fibre visibility. Three sections were randomly chosen from each biopsy, and the average IENFD was calculated.

IENFD measurements were conducted following the guidelines and compared against international reference standards for age and sex [24,25].

### 2.5. Ophthalmological Examination

The ophthalmological exam included uncorrected and best corrected visual acuity, intraocular pressure measures, and a slit-lamp with fundus examination. The ocular surface and tear film were further investigated by the Schirmer I test—evaluated as pathological if 5 or less mm in 5 min; tear break-up time (TBUT)—evaluated as normal if longer than 10 s; and fluorescein staining based on the Oxford scale [26]—evaluated as pathological if it was 2 or more. Corneal sensitivity was tested through semiquantitative evaluation of the corneal reflex with a cotton wool fibre and evaluated as normal, diminished, or absent. The ocular surface was evaluated in both eyes and the worst parameter from either eye was selected for analysis.

To evaluate the possible trophic effect of corneal denervation, specular microscopy of the corneal endothelium was performed with a specular microscope EM-400, Tomey, Nagoya, Japan. Endothelial cell density was analysed in cell/mm^2^.

### 2.6. Corneal In Vivo Confocal Microscopy

Corneal in vivo confocal microscopy (IVCM) was performed with the Heidelberg Retinal Tomograph 3 Rostock cornea module (HRT3 RCM; Heidelberg Engineering, Heidelberg, Germany) within 1 month of skin biopsy.

**Figure 1:**
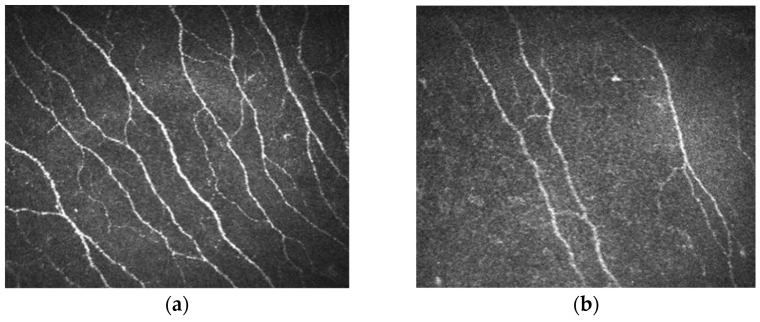
(**a**) Image of a normal corneal sub-basal plexus; (**b**) image of a corneal sub-basal plexus with reduced innervation.

The investigation was performed under topical anaesthesia with benoxinate hydrochloride 0.4% drops. Viscoelastic gel was applied to the tip of the HRT3 RCM lens, and a sterile Tomocap (Heidelberg Engineering, Heidelberg, Germany) was placed over the lens to facilitate optical coupling with the cornea. The patient was asked to focus with the contralateral eye on a target to minimize eye movement during the procedure (Figure 1a). After adjusting for proper depth and focus, a satisfactory number of images were taken of each eye. Three high-quality images from the centre of the cornea, showing the best visibility of the sub-basal nerve plexus, were selected for each eye, following the procedure used to determine the normative values [27]. Images were taken from the layer just behind the basal epithelium and in front of Bowman’s layer. The best-focused, most complete images were selected, ensuring they were in the same layer, free of motion artifacts and folds, and with good contrast (Figure 1b).

Altogether, we analysed six images for each patient. In three cases, where only one eye of the participant fit the inclusion criteria, six images were selected from the one suitable side. To address potential measurement bias, IVCM analysis was performed before accessing the skin biopsy results.

The images were analysed using specialized IVCM software, CCMetrics version 1.1 (University of Manchester, Manchester, UK). The measured parameters included the corneal nerve fibre density (CNFD) in main fibres/mm^2^, representing the number of major nerves per square millimetre; corneal nerve fibre length (CNFL) in mm/mm^2^, representing the total length of all nerve fibres and branches; and corneal nerve branch density (CNBD) in branches/mm^2^, representing the number of branches extending from major nerves. The results were compared to a normative dataset [27]. They were determined as pathological if the parameters were below the 5th percentile for the patient’s age and gender. An alternative method of analysis used the optimal cut-off points determined by Petropoulos [28].

### 2.7. Statistical Analysis

SPSS version 19 (SPSS Inc, Chicago, IL, USA) and Microsoft Excel 2502 (Microsoft, Redmond, WA, USA) were used for statistical analysis. The Shapiro–Wilk test was used to examine the normality of continuous variables. Continuous variables with a normal distribution were expressed as a mean with standard deviation. For proportions, the exact Clopper–Pearson confidence interval was calculated, with 95% confidence intervals. Normally disturbed continuous parameters were tested using unpaired Student’s *t*-test. Categorical variables were compared using the Chi-square test or Fisher’s exact test if cells with expected frequencies below five were present in a contingency table. Cohen ’s Kappa coefficient was used to calculate the effect size for categorical agreement. A *p*-value of <0.05 was considered to be significant.

## 3. Results

**Figure 2:**
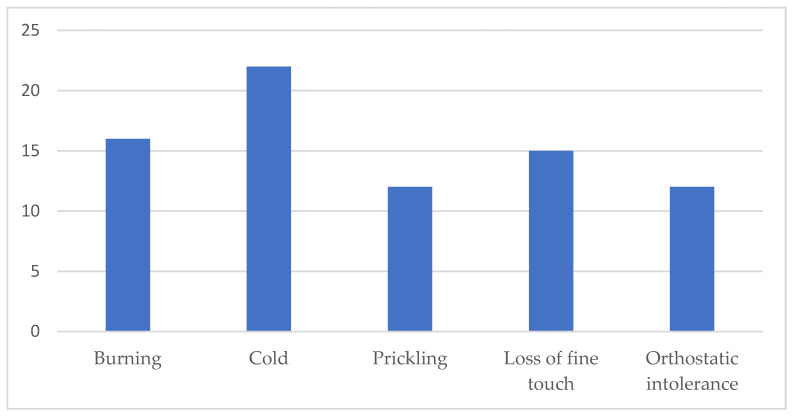
Clinical symptoms of participants, characteristic of small fibre neuropathy.

A total of 36 patients enrolled in the study. One was excluded due to laser corneal correction, leaving 35 patients with clinical suspicion of SFN who were included in the final analysis. The mean age of the participants was 62 years, SD 13.5. There were 11 males and 24 females. The majority of the participants had characteristic length-dependent SFN symptoms (88.6%). The main symptoms are graphically represented in Figure 2.

### 3.1. Skin Biopsy and In Vivo Confocal Microscopy of the Cornea

The skin biopsy proved significantly reduced IENFD (‘positive skin biopsy’) in 14 patients (Table 1)—group 1. The IENFD was normal (‘negative skin biopsy’) in 21 patients—group 2.

The mean CFNL was 15.2, with SD of 3.5 mm/mm^2^. Pathological CFNL below the 5th percentile for age and gender was measured in 13 participants, ranging from 8.8 to 13.5, with a mean of 11.9 and SD of 1.3 mm/mm^2^.

The CNBD was 44.0, SD 17.5 branches/mm^2^. Pathological CNBD below the 5th percentile was measured in three participants, ranging from 14 to 22.3, with a mean of 19 and SD of 7.7 branches/mm^2^.

The mean CNFD was 19.2, with a SD of 3.1 main fibres/mm^2^. Pathological CNFD was measured in one participant, with a value of 13.2 main fibres/mm^2^.

### 3.2. Comparison of Skin Biopsy Results to IVCM Corneal Measurements

Comparison of the IVCM measurements to skin biopsy results is presented in Table 2. Patients with positive skin biopsy in group 1 had a significant rarefication of corneal sub-basal plexus compared to patients in group 2 in terms of significantly reduced CNFL (group 1: 13.667 ± 2.994 vs group 2: 16.272 ± 3.544, *p* = 0.0304) and CNBD (group 1: 36.676 ± 14.681 vs group 2: 48.812 ± 17.830, *p* = 0.0424), while CNFD was not significantly reduced (group 1: 18.472 ± 3.8 vs group 2: 19.719 ± 2.584, *p* = 0.2552).

CNFL was reduced below the 5th percentile in 9 of 14 (64%) patients in group 1 and only in 4 of 21 (19%) patients in group 2. Pathological CNFL thus revealed 64.28% sensitivity (95% CI: 35.1–87.2%) and 80.95% specificity (95% CI: 58.1–94.6%) (*p* = 0.0066) compared to skin biopsy. Cohen’s Kappa coefficient (κ) between CNFL and IENFD was 0.458 (0.156–0.759). The cut-off value of 15.8 mm/mm^2^ determined by Petroupoulos displayed higher sensitivity of 82.4% (95% CI: 56.6–96.2%) and lower specificity of 55.6% (95% CI: 30.8–78.5%), with a κ value of 0.376 (0.083–0.669) [28].

CNBD was below the 5th percentile in three patients in group 2. CNBD below the 5th percentile was not concordant with the skin biopsy results, nor was CNFD below the 5th percentile, which turned out to be below the 5th percentile in one patient in group 1. A CNBD cut-off value of 41.7 branches/mm^2^ produced 73.3% sensitivity (95% CI: 44.9–92.2%) and 70% specificity (95 CI: 45.7–88.1%), with a κ value of 0.426 (0.128–0.725) [28]. A CNFD cut-off value of 18.77 main fibres/mm^2^ produced 64.3% sensitivity (95 CI: 35.1–87.2%) and 61.9% specificity (95% CI: 38.4–81.9%), with a κ value of 0.253 (−0.064–0.569) [28].

### 3.3. Ophthalmological Exam Findings and IVCM Results

The results of the ophthalmological examination for the group of patients with normal CNFL, and CNFL below the 5th percentile are presented in Table 3. The dry eye findings, and corneal endothelial cell density did not correlate to corneal innervation on IVCM.

### 3.4. Clinical Characteristics Compared to IVCM and Skin Biopsy Results

The clinical characteristics of groups 1 and 2 and patients with reduced and normal CNFL are presented in Table 4 and Table 5. There were no significant differences in age, gender, diabetes, presence of length-dependent symptoms, burning, cold, prickling, fine touch, orthostatic intolerance, and corneal sensitivity between the groups. However, dyslipidaemia was more prevalent in group 1 compared to group 2 (8/14 (57.1%) vs. 3/21 (14.3%), *p* = 0.012). On the other hand, there was no statistically significant difference in dyslipidaemia between the patients with reduced and normal CNFL (5/13 (38.5%) vs. 6/22 (27.3%), *p* = 0.475).

### 3.5. Questionnaire Results in Comparison to IVCM and Skin Biopsy Outcomes

The mean scores for the Neuropathic Pain Scale questionnaire and the Orthostatic Hypotension Questionnaire are presented in Table 6 and Table 7. They demonstrate scores for patients with positive or negative skin biopsy results and normal or abnormal CNFL. The results were not significantly different in group 1 (positive skin biopsy) compared to group 2 (negative skin biopsy). Additionally, the NPS and the OHQ were not significantly different between participants with reduced CNFL and normal CNFL.

### 3.6. Comparison of IVCM and Skin Biopsy Results in Secondary and Idiopathic SFN

**Figure 3:**
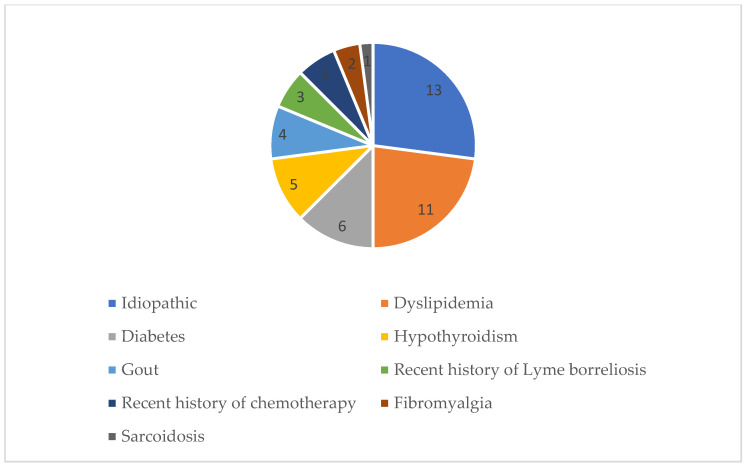
Small fibre neuropathy aetiology.

In more than half of the participants (62.9%, 22 of 35), SFN was proposed to be secondary with different possible causative origin: diabetes mellitus, dyslipidaemia, recent history of chemotherapy, sarcoidosis, fibromyalgia, hypothyroidism, gout, recent Lyme borreliosis (Figure 3).

**Figure 4:**
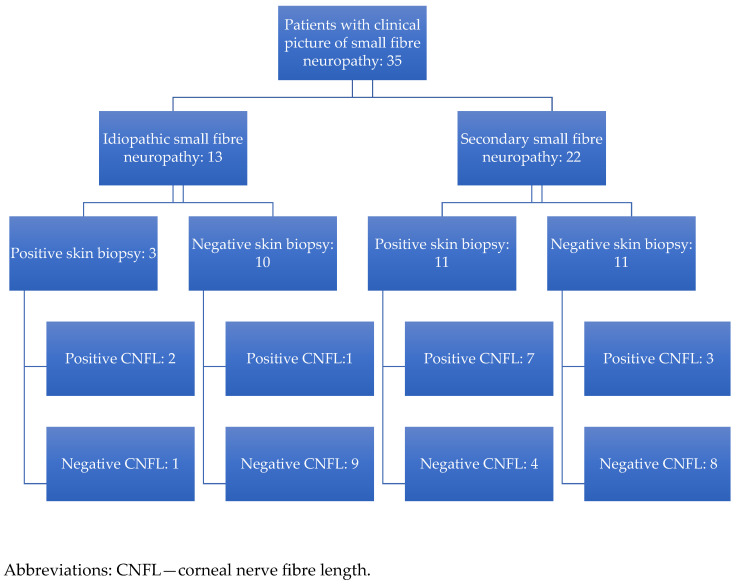
Skin biopsy and corneal nerve fibre density in idiopathic and secondary small nerve fibre neuropathy.

Biopsy and CNFL results in idiopathic and secondary SFN are presented in Figure 4. If skin biopsy results are taken as the gold standard for SFN diagnosis, CNFL would have a positive predictive value (PPV) of 70% (95% CI: 34.8–93.3%; 7 of 10 patients), and a negative predictive value (NPV) of 66.7% (95% CI: 34.9–90.1%; 8 out of 12 patients) in the subgroup with secondary SFN. In the subgroup of idiopathic SFN cases, the NPV was 90% (95% CI: 55.5–99.8%; 9 out of 10 patients).

## 4. Discussion

Our study proves that corneal nerve fibre length in mm/mm^2^ (CNFL) measured by in vivo confocal microscopy of the cornea, with application of a normative database, can separate healthy people from those with SFN with reasonable accuracy, comparable to intraepidermal nerve fibre density (IENFD).

We demonstrated that peripheral neurodegeneration in SFN can occur either simultaneously or in a patchy pattern across various body parts. The former was proven by the correlation between decreased IENFD on skin biopsy and decreased corneal innervation on in vivo confocal microscopy in some but not all patients. The patchy variant of SFN neurodegeneration is the most likely explanation for patients with pathologically decreased CNFL and normal results in skin biopsy. Our findings support the theory that SFN causes systemic but not necessarily simultaneous damage to Aδ and C nerve fibres. Local factors theoretically also influence corneal involvement in SFN and thus explain the variability in denervation patterns between the skin and the cornea.

Cornea could be an ideal area of study for SFN, because it is the most densely innervated tissue in the human body [13,29]. Rich corneal innervation has protective and trophic roles and is also considered a baseline mechanism for maintaining a healthy ocular surface and tear film [30]. Corneal transparency allows for the in vivo detection of pathological changes in corneal nerve fibre, rendering it a unique tissue for investigation of peripheral innervation.

IVCM enables the quantification of small fibres located in the central cornea. It is non-invasive and fast, taking approximately 2 min per eye. A single image covers about 0.16 mm^2^, representing about 0.15% of the total cornea surface. Different studies have proven that 3 [31] to 8 [32] images provide acceptable accuracy compared to a wide-field map of IVCM images. IVCM parameters for evaluation of corneal nerves include (i) corneal nerve fibre density (CNFD), (ii) corneal nerve branch density (CNBD), and (iii) corneal nerve fibre length (CNFL). There is a database of normal values and pathological values (5th percentile) for each parameter [27].

For peripheral neuropathy in patients with diabetes, corneal nerve fibre length (CNFL) on IVCM is accepted as a diagnostic, staging, and follow-up parameter; diagnostic sensitivity and specificity are higher than 90% for diabetic polyneuropathy, and analysis of CNFL has been proposed as the most reliable diagnostic marker for patients with SFN [12,33,34].

Besides at least one SFN symptom and one clinical SFN sign, another confirmatory objective finding is required to establish the diagnosis of SFN, which is most commonly reduced intraepidermal small fibre density [5]. This pathological criterion was reported to be positive in 85% of patients with a clinical picture of SFN [7]. Negative samples in a portion of symptomatic patients are explained by changes in excitability without degeneration—so-called pathologically non-specific SFN [35,36]. Functional tests (Quantitative Sensory Testing, QST), on the other hand, detect functional impairment of sensory nerve fibres (Aδ-fibres, Aβ-fibres, and C-fibres) and rely on the patient’s active and subjective cooperation [37]. However, a study by Galosi demonstrated that functional tests, such as QST, correlated with neuropathic pain and autonomic symptoms, while IENFD did not [38]. Our study is line with these results, as there were no correlations between IENFD or CNFL and the scores of the NPS and OHQ questionnaires. The lack of correlation between the severity of neuropathic pain and autonomic symptoms with morphological nerve changes proves the importance of incorporating functional tests.

Skin biopsy has 90% diagnostic accuracy and is the preferred method to prove SFN, while functional tests have about 50% diagnostic accuracy and serve as a secondary approach, especially in cases without intraepidermal small fibre density changes [6]. A mild or early SFN cannot be ruled out even if the patient does not have a positive biopsy result [39]. An alternative test of autonomic function has been proposed in such circumstances: the quantitative sudomotor axon reflex test (QSART) [40]. This test can diagnose and monitor autonomic dysfunction, which is also supported by small fibres. Unmyelinated and thinly myelinated sympathetic nerve fibres innervate sweat glands. The QSART assesses postganglionic sudomotor function by stimulating unmyelinated C-fibres with acetylcholine and measuring sweat response. The QSART has approximately 50% sensitivity for SFN diagnosis [41]. However, Sudoscan is a more affordable method and offers comparable results to the QSART [42,43,44].

In search of alternative methods with higher sensitivity than sensory or autonomic tests, analysis of corneal innervation by IVCM parameters has been suggested in patients with SFN [45,46,47,48]. IVCM not only enables direct imaging of peripheral nerves, but it does so non-invasively and can be repeated for follow-up of the disease or treatment.

In contrast to previous research by Tavakoli et al., we considered positive results of the golden standard skin biopsy as confirmation of SFN diagnosis, and correlated the IVCM results to skin biopsy results [8,45]. The pathological IVCM parameter findings in our study were compared to the normative dataset for different ages and gender. This was not performed by previous authors, including Bucher et al. and Tavakoli et al. This comparison to a normative dataset allows for precise evaluation and also follows the same concept as used for the evaluation of skin biopsies [25,27,45,46].

Absolute measurements of corneal nerves on IVCM in our patients with reduced intraepidermal small fibre density (IENFD), i.e., positive skin biopsy, demonstrated both significantly reduced CNFL and CNBD. However, after applying the normative values, the results for CNBD were not useful anymore, while CNFL was found to be the optimal IVCM diagnostic parameter for SFN. This finding aligns with the research on diabetic sensorimotor polyneuropathy, where CNFL is regarded as the most stable metric and has been identified as the most reliable parameter for detecting diabetic sensorimotor polyneuropathy [49]. Applying IVCM cut-off points determined by Petropoulos improved the results of CNBD and CNFD as diagnostic parameters [28]. Nevertheless, a CNFL below the 5th percentile was the preferred diagnostic method, since it follows the method also used to define pathologic skin biopsy (below the 5th percentile) and enables age- and gender-adjusted analysis, which also produced slightly superior results.

Compared to Bjørnkaer et al., who established the diagnosis of SFN based on at least two of the following criteria: decreased or absent pinprick on bedside examination, decreased or absent thermal sensation on bedside examination, abnormal QST, abnormal IENFD, or CDT (cold detection threshold) [10], our study employed a more direct comparison of neurodegeneration between the cornea and skin by considering abnormal IENFD on skin biopsy as a necessary criterion of SFN diagnosis. In their study, Bjørnkaer et al. included, among others, 30 patients with pure SFN and showed that the sensitivities of IVCM parameters, IENFD, and CDT to establish the diagnosis of SFN were 0.53, 0.37, and 0.30, respectively [10]. However, the specificity and positive predictive value were lower for IVCM compared with IENFD and did not correlate with neuropathy severity, which led them to conclude that IVCM could not replace skin biopsy [10]. However, the sensitivity of IENFD was much lower than in most other similar studies, which makes one wonder if they had overdiagnosed SFN patients without confirmed morphological changes in peripheral neurodegeneration [6,10].

A number of causative pathological conditions were diagnosed in our patients. Namely, 22 out of 35 cases (Figure 4) turned out to be secondary SFN, with more than one underlying cause found in these patients. The CNFL was above the 5th percentile (negative) in 90% of negative biopsies in idiopathic SFN cases, which suggests that it could be used to reliably rule out SFN in idiopathic cases. There were only three patients with idiopathic SFN with a positive skin biopsy, and of these, the CNFL was positive in two cases. In cases of secondary SFN, the outcome was even more useful because it provided evidence of reduced corneal nerve fibre density in 3 out of 11 patients with negative skin biopsies (27.3%). This finding is in line with studies demonstrating that in diabetic neuropathy and sarcoidosis, IVCM has better sensitivity compared to skin biopsy to detect small fibre abnormalities [16,50]. Excluding six participants with diabetes did not significantly alter the accuracy of IVCM compared to skin biopsy (60% sensitivity (95% CI: 26.2–87.8%) and 84.2% specificity (95% CI: 60.4–96.6%), *p* = 0.0317).

Our study also demonstrated a significant correlation between dyslipidaemia and reduced IENFD on skin biopsy (*p* = 0.0115). This aligns with the finding that dyslipidaemia contributes to the loss of small nerve fibres [51,52,53]. Some reports have suggested that dyslipidaemia might contribute as much as impaired glucose metabolism in the development of peripheral neuropathy [54]. Surprisingly, dyslipidaemia was not associated with decreased CNFL (15.25 ± 3.67 mm/mm^2^ vs. 14.83 ± 3.55 mm/mm^2^, *p* = 0.967) and was not associated with CNFLs below the 5th percentile (*p* = 0.258). Perhaps corneal neurodegeneration is less susceptible to dyslipidaemic factors than the skin. This finding also demonstrates the value of assessment of different body areas for small peripheral neuropathy. Skin biopsy and corneal IVCM enable a small window into the anatomy of small peripheral nerve fibres. Therefore, they represent valuable yet imperfect methods for assessing the state of peripheral neurodegeneration. Future studies comparing skin biopsies and IVCM should focus on different etiological subgroups of SFN to determine the preferred method for detecting peripheral neurodegeneration based on specific aetiology.

A systemic review revealed significant variability in the diagnostic criteria used to identify patients with SFN and found no golden standard for its diagnosis in clinical research [55]. In addition, there is no widely accepted guideline in clinical practice [8]. A possible useful classification could be dividing the disease into (i) a group with confirmed peripheral neurodegeneration based on skin biopsy or IVCM, and (ii) a group with confirmed dysfunction of peripheral neurodegeneration based on a positive QST or QSART.

There are certain limitations in our study. First, we did not include the functional diagnostic methods, such as QST and QSART, so patients with functional dysfunction without morphologic changes were not considered to have confirmed SFN. Second, no correlation could be confirmed between the IENFD or IVCM results and ocular surface tests, which could manifest as a consequence of corneal nerve dysfunction. We suggest future studies with a different design, comparing the ocular surface examinations with peripheral nerve dysfunction observed on QST and QSART to determine if there is a correlation. Another limitation was the manual test of reduced corneal sensitivity. This is the simplest test for corneal sensitivity and could also be performed by neurologists. Out of all performed ophthalmologic tests, this was the most reliable measure of reduced CNFL compared to normal CNFL: 38.5% vs. 13.6%. However, the results are not statistically significant (*p* = 0.116). To attain statistically significant results, a future study should include more participants and perhaps measure corneal sensitivity more precisely using an esthesiometer.

Future studies should encompass larger multicentric cohorts of various SFN aetiologies to determine the utility of IVCM for different subgroups. To make IVCM a standard diagnostic method, such studies would need to validate it in a diverse patient pool. Moreover, they should include functional tests, such as QST or Sudoscan, to also detect the functional impairment of nerve fibres.

## 5. Conclusions

In conclusion, our study demonstrates that IVCM of the cornea complements skin biopsy in demonstrating neurodegeneration in patients with SFN. Since it is a non-invasive and readily repeatable procedure, it could be applied as a primary measure to monitor disease progression and objectively assess the effect of treatment. However, due to the small sample size, additional studies of larger cohorts are needed before implementation into routine clinical practice as a diagnostic procedure for SFN.

## Figures and Tables

**Table 1:** Patients with positive skin biopsy or corneal nerve fibre length (CNFL) on IVCM or both (yellow).

Intra-Epidermal Nerve Fibre Density (mm/mm^2^), Increasing Below the 5th Percentile in Bold	Sex	Age (Years)	CNFL—Corneal Nerve Fibre Length (Nerve Fibres mm/mm^2^), Below 5th Percentile in Bold
**0.2**	m	39	14.3
**0.5**	m	69	**10.2**
**1.2**	m	48	**11.1**
**1.2**	f	57	14.8
**1.6**	m	53	**12.9**
**2.1**	f	75	**8.8**
**2.8**	f	51	20.0
**2.8**	f	74	14.9
**2.9**	f	68	15.8
**3.1**	f	70	**11.6**
3.5	f	50	**11.9**
**3.9**	f	77	**12.9**
**4.0**	f	76	**13.5**
**4.3**	m	76	22.1
**4.3**	f	30	**13.2**
4.9	f	74	**12.9**
5.0	m	75	**11.8**
5.8	f	51	**11.4**
8.2	f	66	**12.2**

Abbreviations: f—female, m—male.

**Table 2:** In vivo confocal microscopy parameters in patients with positive skin biopsy results and negative skin biopsy results.

	Group 1, Positive Skin Biopsy (N = 14)	Group 2, Negative Skin Biopsy (N = 21)	
CNFL (mm/mm^2^)	13.667 ± 2.994	16.272 ± 3.544	***p* = 0.030**
CNFL below 5th percentile	9 (64.3%)	4 (19%)	***p* = 0.007**
CNBD (branches/mm^2^)	36.676 ± 14.681	48.812 ± 17.830	***p* = 0.042**
CNBD below 5th percentile	0	3 (14.3%)	***p* =** 0.259
CNFD (main fibres/mm^2^)	18.472 ± 3.8	19.719 ± 2.584	***p* =** 0.255
CNFD below 5th percentile	1 (7.1%)	0	***p* =** 0.400

Abbreviations: CNFL—corneal nerve fibre length, CNBD—corneal nerve branch density, CNFD—corneal nerve fibre density.

**Table 3:** Comparison of ophthalmological examination results between participants with normal and reduced corneal nerve fibre length.

	CNFL Below the 5th Percentile (N = 13)	CNFL Above the 5th Percentile (N = 22)	
Reduced corneal sensitivity	5 (38.5%)	3 (13.6%)	***p* =** 0.116
Positive Schirmer	5 (38.5%)	9 (40.9%)	***p* =** 0.886
Normal TBUT	2 (15.4%)	3 (13.6%)	***p* =** 1.000
Positive Oxford	3 (23.1%)	5 (22.7%)	***p* =** 1.000
Endothelial cell density (cells/mm^2^)	2496.2 ± 305.5	2460.5 ± 314.8	***p* =** 0.746

Abbreviations: CNFL—corneal nerve fibre length; positive Schirmer—5 s or less; TBUT—tear film break-up time; normal TBUT—longer than 10 s; pathologic Oxford—2 or more.

**Table 4:** Clinical characteristics of patients with positive and negative skin biopsy.

	Group 1, Positive Skin Biopsy (N = 14)	Group 2, Negative Skin Biopsy (N = 21)	
Age (years)	59.4 ± 13.5	63.8 ± 13.5	***p* =** 0.359
Gender			***p* =** 1
Male	4 (28.6%)	7 (33.3%)	
Female	10 (71.4%)	14 (66.7%)	
Diabetes	4 (28.6%)	2 (9.5%)	***p* =** 0.191
Dyslipidaemia	8 (57.1%)	3 (14.3%)	***p* = 0.012**
Length-dependent symptoms	11 (78.6%)	20 (95.8%)	***p* =** 0.132
Burning	7 (50%)	10 (47.6%)	***p* =** 0.890
Cold	9 (64.3%)	13 (61.9%)	***p* =** 0.886
Prickling	4 (28.6%)	9 (42.9%)	***p* =** 0.488
Fine touch	5 35.7%)	10 (47.6%)	***p* =** 0.486
Orthostatic intolerance	4 (28.6%)	9 (42.9%)	***p* =** 0.488
Reduced corneal sensitivity	4 (28.6%)	4 (19%)	***p* =** 0.685

Abbreviations: CNFL—corneal nerve fibre length; CNBD—corneal nerve branch density; CNFD—corneal nerve fibre density.

**Table 5:** Clinical characteristics of patients with normal and reduced CNFL.

	CNFL Below the 5th Percentile (N = 13)	CNFL Above the 5th Percentile (N = 22)	
Age (years)	62.6 ± 14.7	61.7 ± 13.05	***p* =** 0.846
Gender			***p* =** 1.000
Male	4 (30.8%)	7 (31.8%)	
Female	9 (69.2%)	15 (68.2%)	
Diabetes	4 (30.8%)	2 (9.1%)	***p* =** 0.166
Dyslipidaemia	5 (38.5%)	6 (27.3%)	***p* =** 0.475
Length-dependent symptoms	12 (92.3%)	19 (86.3%)	***p* =** 1.000
Burning	4 (30.8%)	13 (59.1%)	***p* =** 0.164
Cold	9 (69.2%)	13 (59.1%)	***p* =** 0.721
Prickling	4 (30.8%)	9 (40.9%)	***p* =** 0.721
Fine touch	3 (23.1%)	12 (54.5%)	***p* =** 0.089
Orthostatic intolerance	3 (23.1%)	10 (47.6%)	***p* =** 0.282
Reduced corneal sensitivity	5 (38.5%)	3 (13.6%)	***p* =** 0.116

Abbreviations: CNFL—corneal nerve fibre length.

**Table 6:** Neuropathic Pain Scale questionnaire and Orthostatic Hypotension Questionnaire scores of participants with positive skin biopsy results and negative skin biopsy results.

	Group 1, Positive Skin Biopsy (n = 14)	Group 2, Negative Skin Biopsy (n = 21)	
Neuropathic Pain Scale questionnaire	38.57 ± 15.84	35.48 ± 18.16	*p* = 0.607
Orthostatic Hypotension Questionnaire	21.64 ± 23.38	25 ± 22.46	*p* = 0.607

**Table 7:** Neuropathic Pain Scale questionnaire and Orthostatic Hypotension Questionnaire scores of participants with normal corneal nerve fibre length and reduced corneal nerve fibre length.

	CNFL Below the 5th Percentile (N = 13)	CNFL Above the 5th Percentile (N = 22)	
Neuropathic Pain Scale questionnaire	37.62 ± 16.07	36.18 ± 18.03	*p* = 0.810
Orthostatic Hypotension Questionnaire	19.38 ± 22.68	26.18 ± 22.61	*p* = 0.397

Abbreviations: CNFL—corneal nerve fibre length.

## Data Availability

The authors will provide raw data supporting the conclusion of this article upon request.

## Supplementary Materials

The following supporting information can be downloaded at: https://www.mdpi.com/article/10.3390/diagnostics15172207/s1, Contains Strobe criteria. The Neuropathic Pain Scale (NPS) questionnaire and the Orthostatic Hypotension Questionnaire (OHQ) can be downloaded at https://www.mdcalc.com/calc/3626/neuropathic-pain-scale-nps; https://heal.nih.gov/files/CDEs/2023-07/ohq-crf.docx (accessed on 1 July 2025).

## Abbreviations

The following abbreviations are used in this manuscript:

CDT	Cold detection threshold
CNBD	Corneal nerve branch density
CNFD	Corneal nerve fibre density
CNFL	Corneal nerve fibre length
HRT3RCM	Heidelberg Retinal Tomograph 3 Rostock cornea module
IENFD	Intraepidermal nerve fibre density
IVCM	In vivo confocal microscopy
NPS	Neuropathic pain score
NPV	Negative predictive value
OHQ	Orthostatic Hypotension Questionnaire
PGP	Protein gene product
PPV	Positive predictive value
QSART	Quantitative sudomotor axon reflex test
QST	Quantitative sensory testing
SD	Standard deviation
SFN	Small fibre neuropathy
TBUT	Tear break-up time
