# Supplementary material for: In Vivo Confocal Microscopy of the Cornea in Diagnosing Small Fibre Neuropathy: A Cross-Sectional Observational Study
Source: Diagnostics (Basel). 2025 Aug 30;15(17):2207. doi: 10.3390/diagnostics15172207 (PMC12427913; doi:10.3390/diagnostics15172207)
Supplement: Supplementary file 1 [file diagnostics-15-02207-s001.zip › diagnostics-3824720-supplementary.pdf]

STROBE Statement—checklist of items that should be included in reports of observational studies

	Item No.	Recommendation	Page No.	Relevant text from manuscript
**Title and abstract**	1	(a) Indicate the study's design with a commonly used term in the title or the abstract	1	Title/Abstract: "A cross-sectional observational study…"
		(b) Provide in the abstract an informative and balanced summary of what was done and what was found	1	Abstract includes background, methods, results with CIs, and conclusions
**Introduction**				
Background/rationale	2	Explain the scientific background and rationale for the investigation being reported	2	Despite encouraging findings in the published literature, the precise diagnostic value of corneal IVCM in SFN patients is still unknown
Objectives	3	State specific objectives, including any prespecified hypotheses	2	The purpose of the study was to assess the diagnostic value of corneal IVCM as compared to the standardised skin biopsy
**Methods**				
Study design	4	Present key elements of study design early in the paper	3	It was a cross-sectional study…
Setting	5	Describe the setting, locations, and relevant dates, including periods of recruitment, exposure, follow-up, and data collection	3	performed from September 2024 to March 2025 at the tertiary Eye Hospital University Medical Centre Ljubljana, Slovenia and

				Institute of Neurophysiology, University Medical Centre Ljubljana, Slovenia.
Participants	6	(a) Cohort study—Give the eligibility criteria, and the sources and methods of selection of participants. Describe methods of follow-up<br>Case-control study—Give the eligibility criteria, and the sources and methods of case ascertainment and control selection. Give the rationale for the choice of cases and controls<br>Cross-sectional study—Give the eligibility criteria, and the sources and methods of selection of participants	3	Inclusion criteria included clinical picture of SFN: at least 1 characteristic symptom (such as burning pain, prickling, sensation of cold or stabbing pain, autonomic dysfunction) and at least 1 positive clinical sign (such as pinprick and thermal hypoesthesia, autonomic skin changes), absence of electrophysiological dysfunction of efferent and large afferent fibres, absence of central nervous system disorders, 18 years or older, performed skin biopsy and were willing to participate in the study and to sign the informed consent. Exclusion criteria included history of ocular trauma, history of corneal surgery or laser corneal prescription correction, history of intraocular surgery, any known ocular disease that

				affects the corneal innervation, such as neurotrophic keratitis, contact lens wear. The invasive nature of the skin biopsy underlined the decision not to perform this in healthy candidates. We, however, compared the symptomatic participants who had negative (normal) sin biopsy results to those with positive (reduced IENFD as compared to the normative database) skin biopsy results.
		(b) Cohort study—For matched studies, give matching criteria and number of exposed and unexposed<br>Case-control study—For matched studies, give matching criteria and the number of controls per case		Not a matched design
Variables	7	Clearly define all outcomes, exposures, predictors, potential confounders, and effect modifiers. Give diagnostic criteria, if applicable	3, 4, 5, 14	The primary outcome was the presence of small fiber neuropathy, assessed by intraepidermal nerve fiber density (IENFD) from skin biopsy and corneal nerve parameters from in vivo confocal microscopy (IVCM). Secondary outcomes included the proportion of participants with reductions

below the 5th percentile of normative values for CNFD, CNFL, and CNBD.

The main exposures/predictors were clinical symptoms of small fiber neuropathy and objective test results (IVCM and skin biopsy). Additional predictors included demographic and clinical variables (age, sex, comorbidities, duration of symptoms). Known factors that may affect corneal nerve morphology, were considered as potential confounders and included in exclusion criteria. Patients with diabetes were excluded in additional analysis.

Age and sex were considered as possible effect modifiers of corneal nerve density with the use age and gender adjusted normative values.

Three sections were randomly chosen from each biopsy, and the average IENFD was calculated. IENFD measurements were conducted following the

				guidelines and compared against international reference standards for age and sex [24,25]. IVCM parameters: They were determined pathological if the parameters were below the 5th percentile for the patient's age and gender. Alternative method of analysis used optimal cut-off points determined by Petropoulos [28].
Data sources/ measurement	8*	For each variable of interest, give sources of data and details of methods of assessment (measurement). Describe comparability of assessment methods if there is more than one group	3,4	Standardised skin biopsy to evaluate the intraepidermal nerve fibre density (IENFD) was performed as part of the diagnostic process in all participants following the European Federation of Neurological Societies and the Peripheral Nerve Society guideline [24]. IENFD was quantified from a 3-mm punch biopsy obtained 10 cm above the lateral malleolus, with local anaesthesia, using a 3 mm disposable punch under sterile technique. Samples

				were immunoassayed with PGP 9.5 antibody to improve the fibre visibility.<br>Corneal In vivo confocal microscopy (IVCM) was performed with the Heidelberg Retinal Tomograph 3 Rostock cornea module (HRT3 RCM; Heidelberg Engineering, Heidelberg, Germany) within 1 month of skin biopsy. The images were analysed using a specialized IVCM software CCMetrics (University of Manchester, Manchester, UK).<br>The participants included in the study were asked to fill out two questionnaires to assess the SFN symptoms: i. Neuropathic Pain Scale (NPS) questionnaire (scale X to Y, higher number indicating more severe pain), and ii. The Orthostatic Hypotension Questionnaire (OHQ)
Bias	9	Describe any efforts to address potential sources of bias	3	To address potential measurement bias, IVCM analysis were performed

				before access to skin biopsy results. To reduce selection bias, all patients were consecutively recruited from the neurology clinic based on predefined criteria. We acknowledge the referral bias, since patients were recruited from a tertiary center, which may not represent the general SFN population.
Study size	10	Explain how the study size was arrived at	3	The sample size reflected all consecutive eligible patients during the study window who agreed to additional ophthalmological exam.
Quantitative variables	11	Explain how quantitative variables were handled in the analyses. If applicable, describe which groupings were chosen and why	5	Shapiro-Wilk Test was used to examine the normality of continuous variables. Normally disturbed continuous parameters were tested using unpaired Student's t-test. Categorical variables were compared using the Chi-Square Test or Fisher's exact test if cells with expected frequencies below five were present in a contingency table.

Statistical methods	12	(a) Describe all statistical methods, including those used to control for confounding	5	For proportions, the exact Clopper-Pearson confidence interval was calculated, with a 95% confidence intervals. . Cohen 's Kappa coefficient was used to calculate the effect size for categorical agreement.
		(b) Describe any methods used to examine subgroups and interactions	10	Biopsy and CNFL results in idiopathic and secondary SFN are presented in Figure 5. If skin biopsy results were taken as the gold standard for SFN diagnosis, CNFL would have a 70% (95% CI: 34.8%-93.3%) (7 of 10 patients) positive predictive value (PPV), and a 66.7% (95% CI: 34.9%-90.1%) (8 out of 12) negative predictive value (NPV) in the subgroup with secondary SFN. In the subgroup of idiopathic SFN cases, the NPV was 90% (95% CI: 55.5%-99.8%) (9 out of 10).
		(c) Explain how missing data were addressed	5	In 3 cases, where only one eye of the participant fit the inclusion criteria, six images were selected from the one suitable side.
		(d) Cohort study—If applicable, explain how loss to follow-up was addressed	3	To reduce selection bias, all patients were consecutively

		Case-control study—If applicable, explain how matching of cases and controls was addressed<br>Cross-sectional study—If applicable, describe analytical methods taking account of sampling strategy		recruited from the neurology clinic based on predefined criteria
		(e) Describe any sensitivity analyses	14	Excluding 6 participants with diabetes did not significantly alter the accuracy of IVCM compared to skin biopsy (60% sensitivity (95% CI: 26.2%-87.8%) and 84.2% specificity (95% CI: 60.4%-96.6%), p=0.0317).
**Results**				
Participants	13*	(a) Report numbers of individuals at each stage of study—eg numbers potentially eligible, examined for eligibility, confirmed eligible, included in the study, completing follow-up, and analysed	5	36 patients enrolled in the study. One was excluded
		(b) Give reasons for non-participation at each stage	5	One was excluded due to laser corneal correction, leaving 35 patients with clinical suspicion of SFN who were included in the final analysis.
		(c) Consider use of a flow diagram		Not used
Descriptive data	14*	(a) Give characteristics of study participants (eg demographic, clinical, social) and information on exposures and potential confounders	5,9	Figure 2, Table 4
		(b) Indicate number of participants with missing data for each variable of interest	5	In 3 cases, where only one eye of the participant fit the inclusion criteria, six images were selected from the one suitable side.
		(c) Cohort study—Summarise follow-up time (eg, average and total amount)		Not a cohort study
Outcome data	15*	Cohort study—Report numbers of outcome events or summary measures over time		

		Case-control study—Report numbers in each exposure category, or summary measures of exposure		
		Cross-sectional study—Report numbers of outcome events or summary measures	6,7	The skin biopsy proved significantly reduced IENFD ('positive skin biopsy') in 14 patients (Table 1) – group 1. The IENFD was normal ('negative skin biopsy') in 21 patients – group 2. CNFL was diminished below the 5th percentile in 9 of 14 (64%) patients in group 1 and only in 4 of 21 (19%) patients in group 2.
Main results	16	(a) Give unadjusted estimates and, if applicable, confounder-adjusted estimates and their precision (eg, 95% confidence interval). Make clear which confounders were adjusted for and why they were included	7, 14	CNFL was diminished below the 5th percentile in 9 of 14 (64%) patients in group 1 and only in 4 of 21 (19%) patients in group 2. Pathological CNFL thus revealed a 64.28% sensitivity (95% CI: 35.1%-87.2%) and 80.95% specificity (95% CI: 58.1%-94.6%) ($p = 0.0066$) compared to skin biopsy. Cohen's Kappa coefficient (κ) between CNFL and IENFD was 0.458 (0.156-0.759). Cut-off value of 15.8 mm/mm^2 determined by Petroupoulos displayed higher sensitivity 82.4% (95% CI: 56.6%–96.2%) and lower specificity 55.6% (95% CI: 30.8%

–78.5%), with κ value of 0.376 (0.083–0.669) [28].

CNBD was below the 5th percentile in 3 patients in group 2. CNBD below the 5th percentile was not concordant with skin biopsy results, nor was CNFD below the 5th percentile , which turned out to be below the 5th percentile in 1 patient in group 1. CNBD cut-off value of 41.7 branches/mm^2 produced 73.3% sensitivity (95% CI: 44.9% – 92.2%) and 70% specificity (95 CI: 45.7%–88.1%), with κ value of 0.426 (0.128–0.725) [53]. CNFD cut-off value 18.77 main fibres/mm^2 produced 64.3% sensitivity (95 CI: 35.1%–87.2%) and 61.9% specificity (95% CI: 38.4%–81.9%) with κ value of 0.253 (-0.064–0.569) [28]. Excluding 6 participants with diabetes did not significantly alter the accuracy of IVCM compared to skin biopsy (60% sensitivity (95% CI: 26.2%-87.8%) and 84.2% specificity (95% CI: 60.4%-96.6%), p=0.0317).

	(b) Report category boundaries when continuous variables were categorized	5,7	The images were analysed using a specialized IVCM software CCMetrics (University of Manchester, Manchester, UK). Measured parameters included corneal nerve fibre density (CNFD) in main fibres/mm^2 – expressing the number of major nerves per square milimetre corneal nerve fibre length (CNFL) in mm/mm^2) – expressing the total length of all nerve fibres and branches; and corneal nerve branch density (CNBD) in branches/mm^2 – expressing the number of branches extending from major nerves. The results were compared to a normative data set [27]. They were determined pathological if the parameters were below the 5th percentile for the patient's age and gender. Alternative method of analysis used optimal cut-off points determined by Petropoulos [28].<br>Cut-off value of 15.8 mm/mm^2 determined by Petroupoulos displayed higher sensitivity 82.4% (95% CI: 56.6%–96.2%) and lower specificity 55.6%

				(95% CI: 30.8% –78.5%), with κ value of 0.376 (0.083–0.669) [28]. CNBD was below the 5th percentile in 3 patients in group 2. CNBD below the 5th percentile was not concordant with skin biopsy results, nor was CNFD below the 5th percentile , which turned out to be below the 5th percentile in 1 patient in group 1. CNBD cut-off value of 41.7 branches/mm^2 produced 73.3% sensitivity (95% CI: 44.9% – 92.2%) and 70% specificity (95 CI: 45.7%–88.1%), with κ value of 0.426 (0.128–0.725) [53]. CNFD cut-off value 18.77 main fibres/mm^2 produced 64.3% sensitivity (95 CI: 35.1%–87.2%) and 61.9% specificity (95% CI: 38.4%–81.9%) with κ value of 0.253 (-0.064–0.569) [28].
		(c) If relevant, consider translating estimates of relative risk into absolute risk for a meaningful time period		Not a risk study
Other analyses	17	Report other analyses done—eg analyses of subgroups and interactions, and sensitivity analyses	10	Biopsy and CNFL results in idiopathic and secondary SFN are presented in Figure 5. If skin biopsy results were taken

				as the gold standard for SFN diagnosis, CNFL would have a 70% (95% CI: 34.8%-93.3%) (7 of 10 patients) positive predictive value (PPV), and a 66.7% (95% CI: 34.9%-90.1%) (8 out of 12) negative predictive value (NPV) in the subgroup with secondary SFN. In the subgroup of idiopathic SFN cases, the NPV was 90% (95% CI: 55.5%-99.8%) (9 out of 10).
**Discussion**				
Key results	18	Summarise key results with reference to study objectives	12	Our study proves that corneal nerve fibre length in mm/mm^2 (CNFL) measured by In vivo confocal microscopy of cornea, with application of normative database, can separate healthy people from those with SFN with reasonable accuracy, comparable to intraepidermal nerve fibre density (IENFD).
Limitations	19	Discuss limitations of the study, taking into account sources of potential bias or imprecision. Discuss both direction and magnitude of any potential bias	14	There are certain limitations in our study. First, we did not include the functional diagnostic methods such as QST and QSART, so patients with functional dysfunction without

morphologic changes were not considered as confirmed SFN. Second, no correlation could be confirmed between IENFD or IVCM results and ocular surface tests, which could manifest as a consequence of corneal nerve dysfunction. We suggest future studies with a different design, comparing the ocular surface examinations with peripheral nerve dysfunction observed on QST and QSART to determine if there is a correlation. Another limitation was the manual test of reduced corneal sensitivity. It is the simplest test of corneal sensitivity and could also be performed by neurologists. Out of all performed ophthalmologic tests, it was the most reliable measure of reduced CNFL compared to normal CNFL: 38.5% vs. 13.6%. However, the results were not statistically significant ($p = 0.116$). To attain statistically significant results, the study should include more participants and perhaps measure corneal sensitivity more precisely using an esthesiometer.

Interpretation	20	Give a cautious overall interpretation of results considering objectives, limitations, multiplicity of analyses, results from similar studies, and other relevant evidence	13	On the contrary to previous research by Tavakoli et al., we considered the positive results of the golden standard skin biopsy as a confirmation of SFN diagnosis, and correlateed the IVCM results to skin biopsy results [8,45]. The pathological IVCM parameters findings in our study were compared to the normative data set for different ages and gender, which was not done by previous authors, Bucher et al. nor Tavakoli et al. This comparison to a normative data set allows for a precise evaluation and also follows the same concept as used for the evaluation of skin biopsies [25,27,45,46]. Compared to Bjørnkaer et al, who established the diagnosis of SFN based on at least two of the following: decreased or absent pinprick bedside, decreased or absent thermal sensation bedside, abnormal QST, abnormal IENFD, or CDT (cold detection threshold) [10], in our study a more direct comparison of neurodegeneration between

				cornea and skin by considering abnormal IENFD on skin biopsy a necessary criterion of SFN diagnosis. In their study Bjørnkaer et al included among others 30 patients with pure SFN and showed that sensitivities of IVCM parameters, IENFD, and CDT to establish the diagnosis of SFN are 0.53, 0.37, and 0.30 respectively [10]. However, specificity and positive predictive value were lower for IVCM compared with IENFD, and did not correlate with neuropathy severity, which led them to conclusion that IVCM could not replace skin biopsy [10]. However, the sensitivity of IENFD was much lower than in most other similar studies, which makes one wonder if they overdiagnoses SFN patients without confirmed morphological changes of peripheral neurodegeneration [6,10].
Generalisability	21	Discuss the generalisability (external validity) of the study results	15	due to the small sample size, additional studies of larger

				cohorts are needed before implementation as a diagnostic procedure for SFN into routine clinical practice.
**Other information**				
Funding	22	Give the source of funding and the role of the funders for the present study and, if applicable, for the original study on which the present article is based	15	This study did not receive any external funding.
